# Differentially Expressed MicroRNAs in Maternal Plasma for the Noninvasive Prenatal Diagnosis of Down Syndrome (Trisomy 21)

**DOI:** 10.1155/2014/402475

**Published:** 2014-11-12

**Authors:** Julian Kamhieh-Milz, Reham Fadl Hassan Moftah, Gürkan Bal, Matthias Futschik, Viktor Sterzer, Omid Khorramshahi, Martin Burow, Gundula Thiel, Annegret Stuke-Sontheimer, Rabih Chaoui, Sundrela Kamhieh-Milz, Abdulgabar Salama

**Affiliations:** ^1^Institute for Transfusion Medicine, Charité University Medicine, Augustenburger Platz 1, 13353 Berlin, Germany; ^2^Research Center for Immune Science (RCIS), Charité University Medicine, Hessische Strasse 3-4, 10115 Berlin, Germany; ^3^Institute for Human Genetics, Charité University Medicine, Augustenburger Platz 1, 13353 Berlin, Germany; ^4^Clinical Pathology Department, Faculty of Medicine, Alexandria University, Alexandria, Egypt; ^5^Systems Biology and Bioinformatics Laboratory, University of Algarve, Campus of Gambelas, 8005-139 Faro, Portugal; ^6^Human Genetics Practice, Friedrichstraße 147, 10117 Berlin, Germany; ^7^Prenatal Diagnosis and Human Genetics Centre, Friedrichstraße 147, 10117 Berlin, Germany

## Abstract

*Objectives*. Most developmental processes are under the control of small regulatory RNAs called microRNAs (miRNAs). We hypothesize that different fetal developmental processes might be reflected by extracellular miRNAs in maternal plasma and may be utilized as biomarkers for the noninvasive prenatal diagnosis of chromosomal aneuploidies. In this proof-of-concept study, we report on the identification of extracellular miRNAs in maternal plasma of Down syndrome (DS) pregnancies. *Methods*. Using high-throughput quantitative PCR (HT-qPCR), 1043 miRNAs were investigated in maternal plasma via comparison of seven DS pregnancies with age and fetal sex matched controls. *Results*. Six hundred and ninety-five miRNAs were identified. Thirty-six significantly differentially expressed mature miRNAs were identified as potential biomarkers. Hierarchical cluster analysis of these miRNAs resulted in the clear discrimination of DS from euploid pregnancies. Gene targets of the differentially expressed miRNAs were enriched in signaling pathways such as mucin type-O-glycans, ECM-receptor interactions, TGF-beta, and endocytosis, which have been previously associated with DS. *Conclusions*. miRNAs are promising and stable biomarkers for a broad range of diseases and may allow a reliable, cost-efficient diagnostic tool for the noninvasive prenatal diagnosis of DS.

## 1. Introduction

Chromosomal aneuploidy is a main cause of human prenatal and postnatal morbidity and mortality and the main genetic cause of mental retardation. The most common aneuploidy amongst live-borns is Trisomy 21, phenotypically manifested as Down syndrome (DS) [1]. Down syndrome occurs in approximately 1 in 700 live births worldwide, with the incidence rising with advanced maternal age [1]. Prenatal testing for trisomy's is currently available to women of advanced maternal age, with ultrasound findings consistent with DS or with previous fetuses afflicted with chromosomal abnormalities [2]. A small but measurable risk of fetal loss is associated with prenatal diagnostic invasive procedures [3]. Therefore, there exists a salient necessity to develop noninvasive aneuploidy screening tests with a high level of sensitivity and a negligible rate of false positives.

The demonstration of the presence of fetal nucleic acids in maternal peripheral blood has raised great interest in the field of noninvasive prenatal diagnostics (NIPD) [4]. Circulating cell-free fetal DNA (ccfDNA) molecules have been detected in maternal plasma from the first trimester onwards, with concentrations that increase with progressing gestational age [5, 6]. Subsequently, placental-specific cell-free mRNAs species have been detected in maternal plasma [7]. Cell-free nucleic acids have led to the development of a number of NIPD tests such as the determination of fetal Rhesus D status in Rhesus D negative women [3, 8, 9], sex-linked diseases [10], and *β*-thalassemia [11]. Furthermore, quantitative aberrations of fetus-derived mRNA transcripts have been shown in conditions such as preeclampsia [12]. These findings suggest that the detection of circulating fetal nucleic acids holds much promise for NIPD [13, 14].

Recent studies on microRNAs (miRNAs) suggest that they might represent yet another class of promising molecular markers [15–17]. miRNAs are a family of small, 18–22-nucleotide-long, non-protein-coding RNAs that have emerged as key posttranscriptional regulators of gene expression [14]. miRNAs interact with their target coding mRNA to inhibit protein synthesis by either degradation of the mRNAs or blocking translation without degrading the targets [18]. In mammals, miRNAs are predicted to control the activity of ~50% of all protein-coding genes, and functional studies indicate that miRNAs participate in the regulation of almost every cellular process investigated [14, 19]. Recent data demonstrate that miRNAs play a fundamental role in diverse biologic and pathologic processes which include cell proliferation, differentiation, apoptosis, animal development, carcinogenesis, cardiovascular disease, and primary muscular disorders [20–22]. miRNAs have been demonstrated to be highly stable in whole blood, making them an ideal candidate for noninvasive diagnostic purposes [23].

The overexpression of 10 miRNAs in the human hippocampus and heart from foetuses with DS, including the HSA-21 encoded miRNAs HSA-MIR-99a, -HSA-let-7c, -HSA-MIR-125b-2, HSA-MIR-155, and HSA-MIR-802, has been reported [24]. In a DS mouse model, HSA-MIR-155 was also found to be overexpressed [25]. However, the results from Kuhn and coworkers have been recently retracted. Based on these findings and prior to the retraction, Kotlabova and colleagues investigated the reliability of these five HSA-21 derived miRNAs and although HSA-MIR-155 was also found to be differentially expressed, they reported that miRNAs do not represent suitable biomarkers for the NIPD of DS [26].

A wide range of organ systems are affected in DS individuals, some being congenital whereas others are progressive and include cardiac malformations, craniofacial and skeletal anomalies, increased frequency of childhood leukemia, varying degrees of intellectual disability, and central nervous system abnormalities [27]. With the assumption that ~50% of all genes are miRNA-controlled [19], we hoped to identify DS-specific miRNA profiles in maternal plasma. High-throughput quantitative PCR (HT-qPCR) was applied for the highly accurate and reproducible quantification of 1043 miRNAs in quadruplicate. For the first time, here we report on a subset miRNAs in maternal plasma for the identification of DS. These findings may have great impact on the NIPD of DS in the future.

*What Is Known/What Is New.* Differentially expressed miRNAs have been previously demonstrated in a Down syndrome (DS) mouse model. Extracellular cell-free miRNAs have been identified resulting in the distinction of DS from euploid fetuses. The identified miRNAs counteract with known DS pathomechanisms reconfirming their specificity for DS.

## 2. Materials and Methods

### 2.1. Study Group and Sample Preparation

Blood samples collected in EDTA were obtained from pregnant women for whom invasive genetic testing was recommended due to increased maternal age or suspicious ultrasound findings. Prenatal ultrasound examination was performed in Berlin at the Prenatal Diagnosis and Human Genetics Centre Friedrichstrasse and at the practice of Dr. Ömer Kilavuz, which included nuchal translucency measurement for the risk calculation of Trisomy 21. Blood withdrawal was performed prior to the invasive procedure. Invasive diagnostics were performed in accordance with routine procedures of the Practice for Human Genetics Friedrichstrasse by conventional chromosome analysis (karyotyping). In this proof-of-concept study, plasma from seven Trisomy 21 pregnancies and seven appropriately matched controls (similar maternal age, identical gestational week and fetal gender) was selected (Table 1). Our study was approved by the ethics committee of the Charité (ethics number EA1/262/10), with all participants providing written consent.

### 2.2. MicroRNA Isolation

Blood samples were processed within 24 h following withdrawal. To harvest cell-free plasma, samples were spun at 2,000 rcf for 5 min. Plasma was then transferred to a clean tube followed by centrifugation at 14,000 rcf for 10 min to remove cell debris. Samples were frozen at −80°C until further use. For HT-qPCR, miRNAs were isolated from 200 *μ*L plasma with the miRNeasy Mini Kit (Qiagen, Hilden, Germany) using a modified protocol. In brief, 750 *μ*L fresh QIAzol master mix (800 *μ*L QIAzol and 1.25 *μ*L 0.8 *μ*g/mL MS2 RNA per sample) was added, followed by incubation at RT for 5 min. Following the addition of 200 *μ*L chloroform, samples were incubated for 2 min at RT, followed by centrifugation for 15 min at 12,000 rcf at 4°C. Further preparation of the miRNA samples was performed according to the manufacturer's instructions. DNase digestion is generally not required since the combined QIAzol and RNeasy technologies efficiently remove most of the DNA without DNase treatment. Concentrations were measured with the Agilent 2100 Bioanalyzer using small RNA chips (Agilent, CA, USA). As human plasma samples contain only low amounts of extracellular cell-free RNA, glycogen (Roche, Germany) was applied as carrier RNA to enhance extraction efficiency and yields.

### 2.3. High-Throughput Quantitative PCR (HT-qPCR)

High-throughput quantitative PCR (HT-qPCR) was performed on individual samples using the SmartChip Human miRNA Panel V3.0 (WaferGen, CA, USA) in accordance with the manufacturer's instructions. The SmartChip Human miRNA Panel V3.0 simultaneously quantitatively measures the expression of the most disease-relevant 1043 human microRNA species in quadruplicate. Polyadenylation of miRNA (100 ng) was performed using a modified protocol of the A-Plus Poly(A) Polymerase Tailing Kit (Epicentre, WI, USA). Polyadenylation was performed by incubation at 37°C for 30 min, followed by a 5 min denaturation step at 70°C. The miRNA templates were reverse transcribed to cDNA using an anchored oligo(dT)-containing primer. RT-PCR was performed by incubating the samples at 40°C for 60 min, followed by a denaturation step for 5 min at 80°C. Quantitative real-time PCRs were performed on SmartChip Human miRNA Panel v3.0 and the SmartChip Cycler with the following thermal profile: polymerase activation for 6 min at 95°C and preamplification for 60 s at 50°C, followed by 39 cycles comprising 95°C for 60 s and 60°C for 70 seconds. A no template control (NTC) was also performed.

### 2.4. Bioinformatical and Statistical Analysis

HT-qPCR analysis was performed using both qBase Software (Biogazelle, Belgium) and BioConductor (HT-qPCR Package). The WaferGen qPCR Software report generated provides a short overview on raw data (replicates) Cqs, distance between sample and NTC, and normalized relative quantities (NRQs). These data were also used as a template for downstream analysis using R and Bioconductor. Data export was recomputed for each sample with the SmartChip qPCR Software to export RMDL data for the qBase Software. This tool which uses individual Cq data and takes into account the flagging system by the SmartCycler software was used for quality control, normalization using the “global mean on common targets,” NTC correction, group comparison, and error propagation. Data analysis was performed using different strategies based on Cq values and normalized relative quantities (NRQs). Cqs values with delta Cqs < 2 were flagged and Cqs values with delta Cqs < 3.3 were termed “marginal.” Additionally, Cq values equaling 0 were flagged. Finally, clustering and classification were performed on standard R functions which were adjusted to the data sets. A *P* value <0.05 was termed as statistically significant.

### 2.5. Predicted miRNA Targets of Differentially Expressed miRNAs

In order to identify predicted miRNA targets, the Diana mirPath tool V2 was used, which also enables follow-up analysis, such as mapping target genes on KEGG pathways. For Diana mirPath database, the default MicroT cut-off value of 0.8 was applied. A merged *P* value is calculated for each pathway by applying Fisher's meta-analysis method. The resulting *P* value depicts the probability that the examined pathway is significantly enriched with gene targets of at least one selected miRNA.

## 3. Results

### 3.1. Identification of Trisomy 21-Associated miRNAs Using HT-qPCR

Seven samples from DS pregnancies and seven matched controls (Table 1) were analyzed in quadruplicate for 1043 mature miRNAs using the WaferGen miRNA SmartChip V3. Three hundred and forty-eight miRNAs which were flagged in all samples were excluded from downstream analysis, resulting in a remaining 695 miRNAs. For statistical analysis, data were filtered to include miRNAs that were identified in at least seven of the 14 samples, remaining in 328 miRNAs. Raw data, normalized to global mean data (GN) as well as quantile normalized data (QN), were investigated on the corrected threshold cycle (Cqs representing Ct values minus background fluorescence of the no template control) and normalized relative quantities (NRQs) for downstream bioinformatical algorithms. Comparison of the relative expression of these miRNAs was performed using different strategies (raw Cqs, GN Cqs, QN Cqs, raw NRQs, and GN NRQs) resulting in the identification of 36 mature miRNAs, which were found to be significantly differentially expressed in DS pregnancies in comparison to the euploid pregnant cohort (Table 2 and Supplement 1 available online at http://dx.doi.org/10.1155/2014/402475). Nine miRNAs were identified as common elements by both Cq and NRQ values, whereas 19 miRNAs were identified only by Cq values and eight only by NRQs.

### 3.2. Identification of Subsets of miRNAs Differentiating between Down Syndrome and Euploid Pregnancies

**Figure 1:**
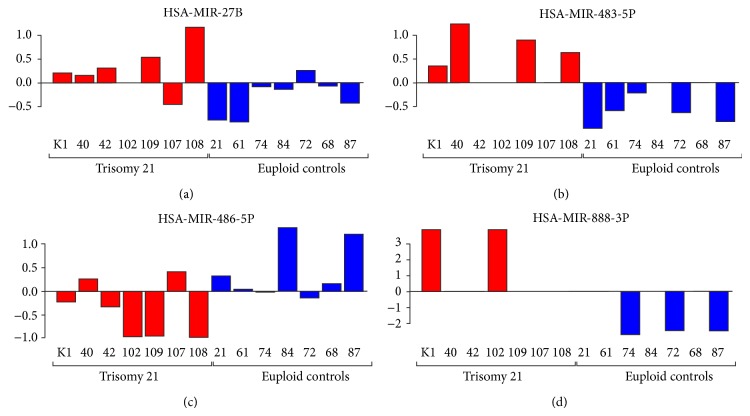
Column plots of selected miRNAs representing an expression profile between Down syndrome versus euploid pregnancies.

**Figure 2:**
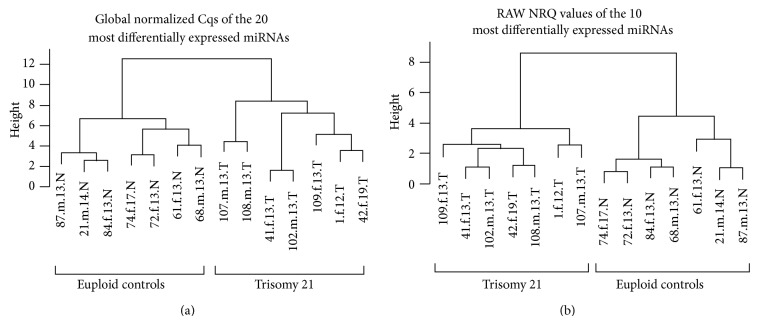
Clustering based on the global normalized Cqs of the 20 most differentially expressed microRNAs (a) and even clustering based on the raw NRQs of only the 10 most differentially expressed miRNAs (b) were performed. As demonstrated here, clustering can be archived that distinguishes Down syndrome from euploid pregnancies utilizing a subset of 10–20 miRNAs. Sample identification comprised of: sample ID number.fetal sex.week of gestation.group. Abbreviations: f = female, m = male, T = Trisomy 21, N = normal.

Although a particular trend of some miRNAs can be observed, a single miRNA that discriminated DS from euploid pregnancies does not exist (Figure 1). Using the full set of miRNAs, a particular miRNA signature allowing for the differentiation between DS and euploid pregnancies was not detected (data not shown). However, by using a subset of 10 or 20 miRNAs that were most significantly differentially expressed in raw NRQs and GN Cqs, respectively, a clear separation of both groups was observed (Figure 2).

### 3.3. MicroRNA Target Prediction

In order to assess our hypothesis that there are distinct differences in the development of DS fetuses which may be reflected by aberrations in the miRNA profile in maternal plasma, miRNA target prediction was performed. Using the Diana mirPath tool (V2), all 36 miRNAs identified by HT-qPCR were annotated in this database. The* a priori* approach (union of genes) revealed the identification of 46 significantly enriched pathways (Supplement 2). In order to increase the stringency of the target pathway prediction, a* posteriori* approach (pathway union) was also applied resulting in the enrichment of the KEGG gene ontology terms mucin type O-glycan biosynthesis, ECM-receptor interactions, glycosaminoglycan biosynthesis (chondroitin sulphate), TGF-*β* signaling, and endocytosis (Table 3, Supplement 2).

## 4. Discussion

A wide range of organ systems are affected in Down syndrome individuals, with some being congenital whereas others are progressive, and include cardiac malformations, increased frequency of childhood leukemia, and varying degrees of mental retardation [27]. miRNAs are considered to play an active role in the regulation of developmental processes. Differentially expressed miRNAs have been previously demonstrated in a DS mouse model [25, 28]. With the assumption that approximately 50% of all genes are miRNA-controlled [19], we were able to identify DS-specific miRNA profiles in maternal plasma (Table 2).

In accordance with Kotlabova and colleagues [26], the miRNAs reported by Kuhn and colleagues (except miR-155) were not found to be differentially expressed in our study. However, developmental dysregulation is rather complex and also involves miRNAs encoded on other chromosomes, thus requiring comprehensive HT qPCR profiling technologies to identify these differences. We identified miR-155 to be differentially expressed, although underrepresented in DS pregnancies in comparison with control cohorts. Hromadníková and colleagues identified this miRNA to be significantly elevated in the plasma of pregnant women compared to nonpregnant controls [29]. A recent study described the identification of miRNA by small RNA sequencing in DS fetuses [30]. Several miRNAs were commonly found including HSA-MIR483-5p, HSA-MIR-486-5p, HSA-MIR-92b, HSA-MIR-181a, and HSA-MIR-155, although expression patterns differed in the first two miRNAs. A direct comparison of both studies is not possible due to numerous differences in the study designs including different input material, sample preparation, clinical numbers, and the application of Next Generation Sequencing (NGS) instead of qPCR-based quantifications. Next Generation Sequencing requires normalization and library preparation steps that alter miRNA profiles, making downstream validation studies using qPCR necessary.

Here we describe the potential use of miRNA patterns for diagnostic purposes. When using 10 or 20 miRNAs that were found to be the most differentially expressed, a clear identification of DS was possible (Figure 2). However, the crosslink between miRNA expression patterns and various pathways that may be dysregulated in DS settings is via the identification of predicted miRNA targets. The advantage of the Diana mirPath tool is that miRNA targets were experimentally validated. An additional distinctive feature of this tool is the follow-up analysis that enables the evaluation of target genes for enrichment amongst KEGG pathways. By evaluating all 36 miRNAs in the Diana mirPath tool, the detected KEGG pathways accurately reflected many known pathomechanisms of DS.

The mucin type O-glycan biosynthesis was found to be the most significantly enriched KEGG pathway using pathway union (Table 2). Human chorionic gonadotropin (HCG) is a glycoprotein hormone produced by placental trophoblasts and trophoblastic tumors [31]. The antibody B152 which mainly recognizes the core-2 O-glycans at Ser-132 has been demonstrated to be useful in the prediction of DS pregnancies [32]. Heat map analysis connecting miRNA with KEGG pathways via the clustering of significance revealed that mucin type O-glycan is closely related to HSA-MIR-498, HSA-MIR-30C-5P, HSA-MIR-629-5P, and HSA-MIR-195-3P. Interestingly, HSA-MIR-498 has been found to be expressed in the human placenta, preferentially in the cytoplasm of syncitiotrophoblasts [33], whereas HSA-MIR-30C has been found in human fetal liver [34], providing additional evidence for a fetal origin of these miRNAs.

The second most significantly enriched GO term identified in this study was ECM-receptor interaction. Grossman and colleagues studied the effect of the coexpression of DSCAM and COL6A2, interaction partners that are overexpressed in DS patients and are associated with cardiomyopathy [35]. In their study, the gene expression profile of cooverexpression versus normal expression also revealed the KEGG gene ontology terms ECM-receptor interaction as the most significant pathway. Furthermore, they identified the GO categories “pathways in cancer,” “arrhythmogenic right ventricular cardiomyopathy,” “regulation of actin cytoskeleton,” “axon guidance,” “dilated cardiomyopathy,” “focal adhesion,” and “hypertrophic cardiomyopathy (HCM),” whereas all these pathways were also identified to be significantly enriched in our study using the “genes union” approach of the Diana mirPath tool (Supplement 2).

Glycosaminoglycans (GAGs) are associated with mental retardation and multiple organ failure and already serve as biomarkers in prenatal diagnostics. This pathway was identified due to the strong linkage of HSA-MIR-362 and HSA-MIR-124. Interestingly, HSA-MIR-362 has been associated with fetal teratogenesis and mental retardation in mice [36], suggesting its relevance in neurogenerative disease. HSA-MIR-124 in addition to HSA-MIR-498, HSA-MIR-20A, and HSA-MIR-629 were found to be associated with DS via a computational approach of human miRNA disease association [37]. Abnormal TGF-*β* has been associated with plaque formation in the brains of Alzheimer's disease and DS patients [38]. Moreover, abnormal TGF-*β* levels have been identified in the amniotic fluid of DS pregnancies [39]. These findings provide evidence that the differentially expressed miRNAs in this study are not randomly identified due to the low sample number but point towards its strong linkage to many well-known DS pathomechanisms.

**Figure 3:**
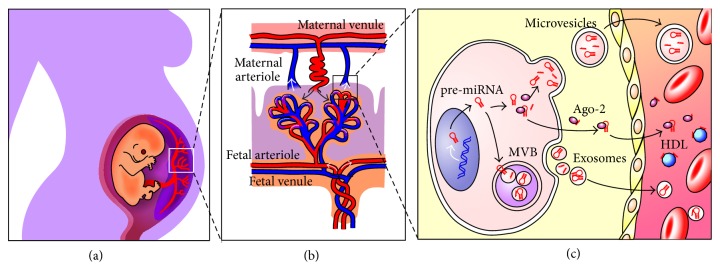
Hypothesized mechanisms on the entrance of fetal/placental miRNAs in the maternal circulation. (a) Connection of the mother and fetus via the placenta. (b) Chorionic villi are responsible for sustaining the placenta with nutrients and oxygen. The intervillus space is filled with maternal blood. (c) Cellular release mechanisms and extracellular transportation systems of miRNAs in accordance with Creemers and colleagues [40]. In the cytoplasm, miRNAs can be incorporated into small vesicles, exosomes, which stem from the endosome, and are released from cells when multivesicular bodies coalesce with the plasma membrane. Microvesicles may also be the source of cytoplasmic miRNAs, which are released from the cell via plasma membrane blebbing. miRNAs are also found in circulation in a microparticle-free form. These miRNAs can be associated with high-density lipoproteins or bound to RNA-binding proteins such as Ago2. It remains unknown as to how these miRNA-protein complexes are released from the cell. They have been suggested to be passively released, as by-products of dead cells, or in an active miRNA-specific manner, via interaction with specific membrane channels or proteins.

Although differential expression patterns of miRNAs can be observed between DS and euploid pregnancies, the exact transport mechanisms of extracellular, cell-free miRNAs into the maternal circulation remain unknown. Several possible mechanisms have been described [40] (Figure 3), although the underlying reason for the release of Trisomy 21-specific miRNAs remains speculative. Trisomy 21 leads to a dysregulation of gene expression including miRNAs. These miRNAs may then enhance the dysregulation of genes in a genome-wide manner, resulting in mild or severe symptoms, depending on the dysregulation. Cells have been demonstrated to select some miRNAs for cellular release while others are retained [41]. Their exocytosis may represent a protective mechanism in which harmful miRNAs are actively enveloped and secreted out of the cells via exosomes. The Down syndrome-specific miRNA profile may therefore reflect the severity of symptoms, for example, in respect to mental retardation, thus classifying miRNAs as the first prognostic biomarkers in the NIPD of DS. In contrast to cffDNA, which is derived from apoptotic placental cells, the miRNA found in maternal plasma may be of fetal and not of placental origin. This hypothesis, however, awaits confirmation.

Much more effort is required to explore miRNA functions in DS. Results demonstrated here have to be confirmed with a broader cohort to validate the usefulness of miRNAs for robust noninvasive diagnostic testing. Furthermore, miRNAs may also allow for antagomiR strategies to cure or at least partially reduce serious DS symptoms during prenatal and postnatal development of DS patients. This may result in a reduction of pregnancy terminations.

Whole and massively parallel genome sequencing have been applied for the detection of DS [42–46]. In contrast to cffDNA and in combination with NGS, our method provides an inexpensive and fast alternative, whereas only chromosomal aneuploidies are investigated. Furthermore, results are readily available within two days. Another advantage is that a diagnostic test based on qPCR can be developed and distributed in the form of a kit. We hope that a diagnostic test based on miRNAs will be relatively less expensive and covered by health insurance companies for all women who would like to obtain a sense of security, which affirms the well-being of the unborn child.

Although miRNAs are considered as promising biomarkers not only for prenatal diagnostics, alterations of miRNA expression during pregnancy and other potential pregnancy-associated complications such as preeclampsia, pregnancy-associated diabetes, and other maternal diseases should also be taken into consideration if miRNA patterns are to be used to identify chromosomal aneuploidies. The use of extracellular cell-free miRNAs for diagnostic purposes may pose a common problem where particular miRNA biomarkers are not present in all samples (Figure 1). This significantly burdens data analysis. In order to stringent statistical analysis, only miRNAs that have been found in at least seven of the 14 samples were used revealing the loss of half of the identified miRNAs. Additionally, the quantitative differences of certain miRNA biomarkers in plasma samples when comparing healthy with disease samples are very low.

In conclusion, extracellular cell-free miRNAs have been identified resulting in the distinction of DS from euploid fetuses. The identified miRNAs counteract with known DS pathomechanisms reconfirming their specificity for DS. miRNAs are highly stable in blood and exhibit potential use for NIPD of DS. Furthermore, miRNAs may represent prognostic markers for the characterisation of the severity of DS disabilities and may allow for antagoMir strategies in “treating” DS features in the future. Due to the low sample number in this proof-of-concept study, these findings can only be considered as promising and as an avenue for further research.

## Supplementary Material

Supplement 1 provides an overview of the HT-qPCR results and bioinformatical analysis.

## Figures and Tables

**Table 1:** Study design and patient characteristics.

Sample	Group	Maternal age [years]	Week of gestation	Fetal gender	Additional notes
1	Trisomy 21 fetuses	36	12 + 1	Female	increased NT
2		43	13 + 3	Female	NT 5.1
3		35	19 + 0	Female	AV canal
4		37	13 + 3	Male	NT 4.2
5		34	13 + 3	Male	NT 7.5, hem
6		42	13 + 4	Male	NT 6.6
7		44	13 + 6	Female	NT 6.6, Softmarker

Matches to 1	Euploid fetuses	42	13 + 1	Female	—
Matches to 2		41	13 + 0	Female	—
Matches to 3		42	17 + 0	Female	—
Matches to 4		40	13 + 3	Male	—
Matches to 5		31	13 + 3	Male	—
Matches to 6		41	14 + 2	Male	—
Matches to 7		35	13 + 5	Female	—

Next Generation Sequencing was performed on pooled samples in order to identify all miRNAs that could be of importance, whereas samples were subsequently individually investigated by HT-qPCR.

Abbreviations: NT: nuchal translucency; AV canal: atrioventricular canal defect; hem: hemolytic plasma.

**Table 2:** List of 36 potential biomarkers identified to be significantly differentially expressed by HT-qPCR.

Number		MicroRNA	Mean Ct of DS	Mean Ct of control	ΔCts	Expression in DS relative to control	*P* value
1	Commonly identified by Cqs and NRQs	HSA-MIR-512-5P	30.97	33.66	−2.69	Overrepresented	0.00861
2		HSA-MIR-483-5P	31.80	33.82	−2.02	Overrepresented	0.00454
3		HSA-MIR-888-3P	30.58	34.67	−4.09	Overrepresented	0.00428
4		HSA-MIR-3676-3P	33.30	31.23	2.08	Underrepresented	0.03754
5		HSA-MIR-3918	27.66	28.39	−0.73	Overrepresented	0.02360
6		HSA-MIR-500A-5P	30.58	31.40	−0.82	Overrepresented	0.02986
7		HSA-MIR-30C-5P	27.05	28.07	−1.02	Overrepresented	0.04203
8		HSA-MIR-200C-3P	32.21	30.53	1.69	Underrepresented	0.04393
9		HSA-MIR-627	33.36	31.97	1.40	Underrepresented	0.04518

10	Identified by Cqs only	HSA-MIR-5893P	31.40	33.86	−2.46	Overrepresented	0.00283
11		HSA-MIR-181A-3P	33.55	30.79	2.75	Underrepresented	0.01864
12		HSA-MIR-362-5P	31.94	34.54	−2.60	Overrepresented	0.02495
13		HSA-MIR-518C-5P	31.35	35.04	−3.69	Overrepresented	0.01990
14		HSA-MIR-3667-5P	30.69	33.21	−2.52	Overrepresented	0.02596
15		HSA-MIR-195-3P	29.35	30.58	−1.23	Overrepresented	0.03372
16		HSA-MIR-124-5P	31.72	33.32	−1.60	Overrepresented	0.03635
17		HSA-MIR-498	34.42	31.45	2.98	Underrepresented	0.02934
18		HSA-MIR-18B-3P	30.71	33.30	−2.59	Overrepresented	0.04078
19		HSA-MIR-1249	26.72	29.20	−2.48	Overrepresented	0.04587
20		HSA-MIR-93-5P	25.76	24.76	1.00	Underrepresented	0.00944
21		HSA-MIR-20A-5P	25.04	24.27	0.77	Underrepresented	0.01837
22		HSA-MIR-4328	32.22	31.25	0.97	Underrepresented	0.02078
23		HSA-MIR-92A-3P	22.95	22.10	0.85	Underrepresented	0.02647
24		HSA-MIR-92B-3P	27.65	26.78	0.88	Underrepresented	0.02926
25		HSA-MIR-3652	30.62	31.64	−1.02	Overrepresented	0.02994
26		HSA-MIR-25-3P	24.19	23.42	0.77	Underrepresented	0.03759
27		HSA-MIR-623	33.77	31.63	2.13	Underrepresented	0.03945
28		HSA-MIR-3195	23.11	24.60	−1.49	Overrepresented	0.04885

29	Identified by NRQs only	HSA-MIR-423-5P	0.87	1.22	−0.35	Underrepresented	0.02007
30		HSA-MIR-27B-3P	1.41	0.85	0.57	Overrepresented	0.03759
31		HSA-MIR-3135a	1.56	0.66	0.91	Overrepresented	0.04044
32		HSA-MIR-486-5P	0.83	1.66	−0.83	Underrepresented	0.02598
33		HSA-MIR-3180-5P	0.71	1.17	−0.46	Underrepresented	0.04036
34		HSA-MIR-629-5P	0.81	1.62	−0.81	Underrepresented	0.04457
35		HSA-MIR-155-5P	0.93	1.89	−0.96	Underrepresented	0.04712
36		HSA-MIR-3907	0.80	1.46	−0.65	Underrepresented	0.04983

NRQs: normalized relative quantities; Cq: Ct values subtracted by background; GN: raw data, normalized to global mean data; QN: quantile normalized data; DS: Down syndrome.

**Table 3:** Gene ontology categories of predicted miRNA targets (pathway union).

KEGG pathway	*P* value	# genes	# miRNAs
Mucin type O-glycan biosynthesis	4.06*E* − 13	8	4
ECM-receptor interaction	1.55*E* − 09	16	7
Glycosaminoglycan biosynthesis-chondroitin sulphate	9.73*E* − 05	2	2
TGF-beta signaling pathway	0.00039237	30	8
Endocytosis	0.00145435	49	6

Presented are KEGG GO terms for the 36 differentially expressed mature miRNAs identified by HT-qPCR (Table 2). Here, the results of pathway union are presented. The full list of GO terms identified by at least one miRNA is presented in Supplement 2. Pathways that have already been previously associated with Down syndrome pathologies were identified. These primarily include the mucin type O-glycan biosynthesis (related to *β*-HCG), ECM-receptor interactions, and TGF-*β* signaling.

#: number of.
